# Complete mitochondrial genome sequence of an Australian little penguin (*Eudyptula minor novaehollandia,* J.R. Forster, 1781)

**DOI:** 10.1080/23802359.2017.1357446

**Published:** 2017-07-27

**Authors:** Subir Sarker, Shubhagata Das, Sarah Frith, Jade K. Forwood, Karla Helbig, Shane R. Raidal

**Affiliations:** a Department of Physiology, Anatomy and Microbiology, School of Life Sciences, La Trobe University, Melbourne, Australia;; b School of Animal and Veterinary Sciences, Faculty of Science, Charles Sturt University, Wagga Wagga, Australia;; c Melbourne Zoo, Parkville, Australia;; d School of Biomedical Sciences, Charles Sturt University, Wagga Wagga, Australia

**Keywords:** Avian mtDNA, mitogenome phylogeny, family Spheniscidae, *Eudyptula minor novaehollandia*

## Abstract

In this paper, we report the complete mitochondrial genome of an Australian little penguin (*Eudyptula minor novaehollandia*). The mitogenome sequence has been found to be circular, and 17,608 bp in length. Similar to other *Spheniscidae* species, the genome encoded a typically conserved structure consisting of 13 protein-coding genes (PCGs), two rRNA genes, and 22 tRNA genes, as well as all protein coding sequences started with methionine. The lengths of 12S ribosomal RNA and 16S ribosomal RNA were 977 bp and 1612 bp, respectively, and were located between tRNA-Phe and tRNA-Leu. The overall base composition of the mitogenome of *E. minor novaehollandia* had slightly higher AT (55.5%) content than GC (45.5%). The complete mitogenome sequence determined in this study would be useful to track the deeper evolutionary history and conservation of *E. minor novaehollandia*.

The Australian little penguin (*E. minor novaehollandia*) is the smallest species of penguin in the family *Spheniscidae* native to coastlines of southern Australia. There are six recognized subspecies with *E. m. novaehollandia* geographically located in Australia. The other five subspecies, *E. m. iredaei*, *E. m. variabilis*, *E. m. albosignata, E. m. minor*, and *E. m. chathamensis* were distributed around the islands of New Zealand. A well-resolved avian phylogenetic tree is required for understanding biogeographic evolutionary structure, whilst there are still major uncertainties in the position of many avian species due to a lack of abundant mitochondrial (mt) datasets. The complete mitogenomes could play a major role to understand the origin, evolution, and divergence time of speciation, as well as influencing conservation and management decisions (Eo et al. 2010). Here, we report a complete mitogenome of *E. minor novaehollandia* for the first time to highlight the species diversity, host phylogeny and ecological diversity of the species.

The blood sample used in this study was obtained from an Australian little penguin in the wild (year of sampling: 2017; GPS location: 37°47′2.886″S, 144°57′5.5692″E), and stored in appropriate condition by the Veterinary Diagnostic Laboratory (VDL), Charles Sturt University under the accession number CS17-0436. Animal sampling was obtained in accordance with approved guidelines set by the Australian Code of Practice for the Care and Use of Animals for Scientific Purposes (1997) and approved by the Charles Sturt University Animal Ethics Committee (Research Authority permit 09/046), and the total genomic DNA was extracted using an established protocol (Sarker et al. 2015, 2016). The genomic library was prepared with an insert size of 150 paired end. A HiSeq2500 sequencing platform (Illumina, Novogene, Nanjing, China) generated approximately 6.77 million sequence reads from the genomic DNA of Australian little penguin. The raw datasets were trimmed to pass the quality control based on PHRED score or per base sequence quality score, and the assembly of the mt genome was conducted according to the established pipeline in CLC Genomics workbench 9.5.4 under La Trobe University Genomics Platform (Sarker et al. 2017). Annotation was performed with MITOS (Bernt et al. 2013), and protein coding ORFs were further assessed using the CLC Genomics Workbench (version 9.5.4).

The complete mt genome sequence of *E. minor novaehollandia* had a circular genome of 17,608 bp, containing 13 protein-coding genes (PCGs), two rRNA genes, and 22 tRNA genes. The contents of A, T, C, and G were 31.0%, 23.5%, 32.0%, and 13.5, respectively. AT and GC contents of this mt genome were 55.5% and 45.5%, respectively. The proportion of coding sequences with a total length of 11,289 bp (64.11%), which encodes 3763 amino acids, and all PCGs started with Met. The lengths of 12S ribosomal RNA and 16S ribosomal RNA were 977 bp and 1612 bp, respectively, and were located between tRNA-Phe and tRNA-Leu. The gene arrangement was similar to the complete mt genome of other *Spheniscidae* species.

Phylogenetic analysis was performed using complete mt genome sequence of *E. minor novaehollandia* determined in this study with the other species belonging to the family *Spheniscidae* available in GenBank. The sequences were aligned using the MAFFT L-INS-i algorithm (Katoh et al. 2002), and the maximum likelihood (ML) tree with 1000 non-parametric bootstrap resamplings were generated using CLC Genomics workbench 9.5.4. As highlighted in Figure 1, the mitogenome from the Australian little penguin was clustered closely to blue little penguin (*E. minor*) native to the New Zealand (Slack et al. 2003), and they also shared >96% pairwise nucleotide identity. We concluded that the complete mitogenome of *E. minor novaehollandia* will be a useful database among the genus *Eudyptula* to study further host-phylogenetic relationship of *Spheniscidae* species, and suggest this may be an implication for the conservation of the species.

**Figure 1.**
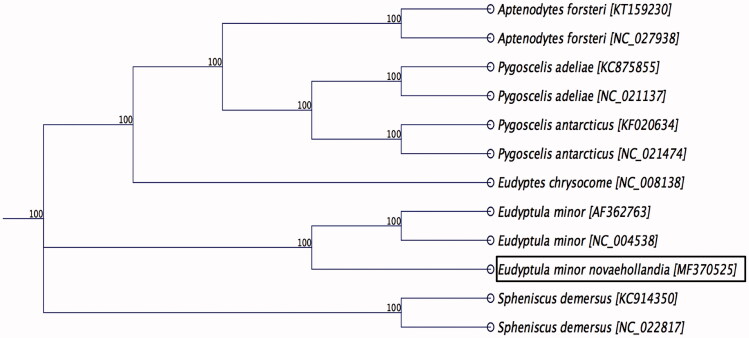
Maximum likelihood phylogenetic tree to infer host-phylogeny relationship among *Spheniscidae* family. ML-tree was constructed using complete mitochondrial genome sequences of the species belonging to the *Spheniscidae* family. The new complete mitochondrial genome of *E. minor novaehollandia* is highlighted by box.
